# Public Attitudes Toward Precision Medicine: A Nationwide Survey on Developing a National Cohort Program for Citizen Participation in the Republic of Korea

**DOI:** 10.3389/fgene.2020.00283

**Published:** 2020-05-12

**Authors:** Hannah Kim, Hye Ryun Kim, Sumin Kim, Eugene Kim, So Yoon Kim, Hyun-Young Park

**Affiliations:** ^1^Division of Medical Law and Ethics, College of Medicine, Yonsei University, Seoul, South Korea; ^2^Asian Institute of Bioethics and Health Law, Yonsei University, Seoul, South Korea; ^3^National Biobank of Korea, Center for Genome Science, Korea National Institute of Health, Cheonju-si, South Korea

**Keywords:** precision medicine cohorts modeling, participant engagement, public attitude, ELSI, benefits for participation, data sharing

## Abstract

This nation-wide survey was conducted among Korean adults to examine the public interest in and attitudes toward establishing a citizen participation cohort model and to collect data to support and determine the future policy and research directions of the Resource Collection Project for Precision Medicine Research (RCP-PMR) before the project proceeds. The demographic framework of the survey population was established based on the statistical standards of the Ministry of the Interior and Safety. An online survey was carried out using web panels between 14 May 2018 and 23 May 2018. Sampling was performed using a simple proportional allocation method considering region, gender, and age. From this survey, the RCP-PMR received very high support (94.5%) and the intention to participate was as high as 83.5%. Respondents had a very positive attitude toward providing their samples and information to the study (84.5–89.9%). In terms of incentives to participate, respondents wanted to receive health information (80.2%), monetary compensation (51.4%), and smart devices (41.3%). Most participants responded that it was appropriate to carry out the project at governmental research institutes (66.9%). Respondents also had a positive attitude toward sharing their information and samples as long as it was only shared with the governmental researchers who run the project (88.0%). However, the survey participants expressed concerns about the study being time consuming or a hassle (38.1%), privacy breaches (33.6%), and the lack of returning benefits of participation (25.1%). Participants had a negative attitude toward sharing their data with researchers who are not directly involved in the RCP-PMR. Considering the future use of the database derived from this project, it will be important to communicate with the lay public as well as the RCP-PMR participants to understand their needs in participating in the forthcoming study and to improve their understanding of the goals of the project, and how data sharing can contribute to disease research and prevention. The RCP-PMR should consider building an efficient citizen-participation program and privacy protection for the research participants.

## Introduction

Precision medicine aims to understand how a person’s genetics, environment, and lifestyle can help determine the best approach to prevent or treat diseases (Collins and Varmus, 2015; Alzu’bi et al., 2019; Genetics Home Reference, 2019). Precision medicine integrates advanced technologies with enriched biomedical big data, including multi-omics; physiological, clinical, mobile, and remote health; and external environmental information to provide transformed healthcare services to one or more people (Collins and Varmus, 2015). For instance, the Precision Medicine Initiative All of Us Research Program (PMI-AURP) in the United States collects specimens and a wide range of personal health information including clinical data, genomic data, and lifelog data from at least 1 million Americans (National Institutes of Health, 2019). The 100,000 Genomes Project funded by the National Institute for Health Research and NHS England involves sequencing 100,000 genomes, including genomic, phenotypic, and other clinical data, from 85,000 patients with rare diseases or cancer (Peplow, 2016; Haga, 2017; Genomics England, 2019). These large-scale precision medicine cohort models necessitate public participation and collective engagement in conjunction with longitudinal collection, access, and use of data (Kaufman et al., 2016).

The Korea Centers for Disease Control & Prevention (KCDC) is planning to carry out the Resource Collection Project for Precision Medicine Research (RCP-PMR) from 2020 onward. This project is expected to collect clinical information, specimens, genetic data, environmental information, and lifelog data – which are essential for research and technological development – from individuals who agree to participate in the RCP-PMR. The collection, storage, and sharing of individuals’ data are expected to be conducted mainly under the *Personal Information Protection Act* and the *Bioethics and Safety Act* (Kim et al., 2018). Qualified researchers who obtain approval to access the database by a proper authority will be able to use the information to conduct a variety of biomedical studies.

However, public acceptance of building a citizen participation national cohort model has not yet been studied in the Republic of Korea (ROK). In the case of the United States large-scale prospective cohort, the nationwide precision medicine initiative cohort study conducted surveys of United States adults to identify public concerns and problems that had to be addressed before the study (Kaufman et al., 2016; Okita et al., 2018). To benchmark a precision medicine cohort program such as the PMI-AURP, we conducted an Ethical, Legal, and Social Implications (ELSI) study in the form of a nationwide survey to confirm public attitude toward precision medicine and to collect opinions on the RCP-PMR before implementing it. The survey identified the social acceptance of the specimen and information provisions and the issues that must be addressed before the project can proceed.

## Materials and Methods

### Survey Methods

We conducted online surveys to collect basic data for future policy directions and research by confirming public attitudes toward and opinions on the RCP-PMR. The online survey participants were recruited based on the statistics of the resident registration of the Ministry of the Interior and Safety at the end of January 2018 (men and women aged 20 and older). Sampling was performed using a simple proportional allocation method considering region, gender, and age. The sample selection and online administration of the survey were managed by the Nielsen Korea online survey firm. During the field period, 1,500 potential respondents of at least 20 years old were randomly sampled from Nielsen’s web-enabled master panel of 500,000 Korean residents. The survey was fielded online between 14 May 2018 and 23 May 2018 (10 days).

### Questionnaire Development

The questionnaire used in this study was developed in reference to the Kaufman et al. survey (Kaufman et al., 2016). The questionnaire was written in simple Korean and included 17 carefully selected multiple-choice questions about the RCP-PMR and eight items on social/demographic variables. The KCDC, which leads this precision medicine national cohort program, provided the draft description of precision medicine, and the RCP-PMR and the authors of this paper completed the description by including a comparison to the PMI-AURP. Respondents answered questions about precision medicine awareness and then confirmed a brief description of precision medicine and the RCP-PMR. Respondents were then asked several questions about the need for the project, their concerns and willingness to participate in the project, and the use of their data. See the questionnaire in the Supplementary Material. After completing the survey, participants received 4,000 South Korean Won (equivalent of USD 3.50) for their time.

### Ethics Approval

The survey was approved by the Institutional Review Board (IRB) of the Yonsei University (approval number: Y-2018-0039). Under the Bioethics and Safety Act, written consent was exempted by the judge of the IRB because, due to the nature of the survey form, the respondents should read the survey information before starting the survey and thus were perceived to have agreed to participate in the survey. The survey was also designed for participants to withdraw their own participation at any time during or after the survey.

### Statistical Analysis

Data were analyzed using the IBM SPSS 20 statistical software. Missing data were excluded by this online survey design. Respondents’ socio-demographic characteristics were analyzed using the variables of gender, age, region, household income, and education (Table 1). In addition, two multiple logistic regressions were examined (Table 2). The attitude toward the RCP-PMR and willingness to participate in the project were the dependent variables.

**TABLE 1:** Respondents’ characteristics.

Variables		*N* (%)
Gender	Men	743 (49.5)
	Women	757 (50.5)
Age	20–29	261 (17.4)
	30–39	259 (17.3)
	40–49	305 (20.3)
	50–59	389 (25.9)
	60+	286 (19.1)
Household Income (KRW)	<\2,000,000	153 (10.2)
	\2,000,000–\3,990,000	458 (30.5)
	\4,000,000–\5,990,000	508 (33.9)
	\6,000,000≤	381 (25.4)
Education	Less than middle school	46 (3.1)
	High school	342 (22.8)
	College and more	1,112 (74.1)
Social networking service	No use	138 (9.2)
	Former use	221 (14.7)
	Current use	1,141 (76.1)

**TABLE 2:** Results of two multiple logistic regressions examining socio-demographic variables related to survey participants’ attitude toward the RCP-PMR and their willingness to participate in the project (*n* = 1,500).

	Demographic Group	Unweighted *N* (weighted percent)	% who said the project definitely or probably should be done	Beta	SE	*p*-value	% who are definitely or probably willing to participate in the project	Beta	SE	*p*-value
Total		1,500 (100)	94.5				83.5			
Gender	Men	743 (49.5)	95.0	0.197	0.234	0.401	87.6	0.641	0.149	0
	Women	757 (50.5)	94.1	ref			79.5	ref		
Age	20–29	261 (17.4)	92.7	−0.810	0.334	0.015	78.5	−0.695	0.207	0.001
	30–39	259 (17.3)	94.6	−0.474	0.359	0.186	79.9	−0.649	0.208	0.002
	40–49	305 (20.3)	93.4	−0.655	0.32	0.041	83.6	−0.404	0.203	0.047
	50+	675 (45.0)	95.7	ref			86.8	ref		
Household	<\2,000,000	153 (10.2)	90.8	−0.659	0.421	0.117	69.3	−1.311	0.263	<0.0001
Income	\2,000,000–\3,990,000	458 (30.5)	94.5	−0.313	0.353	0.376	79.9	−0.805	0.217	<0.0001
	\4,000,000–\5,990,000	508 (33.9)	94.3	−0.412	0.337	0.222	86	−0.386	0.219	0.078
	\6,000,000 ≤	381 (25.4)	96.3	ref			90.3	ref		
Education	Less than middle school	46 (3.1)	84.8	−1.155	0.5	0.021	76.1	−0.272	0.393	0.489
	High school	342 (22.8)	95.6	0.249	0.318	0.434	81	−0.066	0.181	0.716
	College and more	1,112 (74.1)	94.6	ref			84.6	ref		
Social	No use	138 (9.2)	95.3	−0.792	0.338	0.019	84.8	−0.483	0.234	0.039
networking	Former use	221 (14.7)	93.7	−0.270	0.314	0.390	81	−0.215	0.196	0.274
service	Current use	1,141 (76.1)	89.9	ref			76.8	ref		

## Results

### The Respondents

A total of 52,000 people were invited to participate in the survey *via* email and 4,271 connected to the website. Among them, 1,500 people fully responded, resulting in an invitation-response rate of 2.9% and an access-response rate of 35.1%. The demographic characteristics of the survey population are shown in Table 1. The gender distribution was nearly equal, 50.5% female and 49.5% male. The age ranges were 50–59 (25.9%), 40–49 (20.3%), 60+ (19.1%), 20–29 (17.4%), and 30–39 (17.3%). The distribution of household income per month in Korean Won (KRW) was <\2,000,000 (10.2%), \2,000,000 – \3,990,000 (30.5%), \4,000,000 – \5,990,000 (33.9%), and \6,000,000 ≤ (25.4%). The education level ranges were less than middle school graduate (3.1%), high school graduate (22.8%), and college and more (74.1%). Experience with social networking sites (SNS), such as Facebook, Twitter, Instagram, and KakaoTalk, were no use (9.2%), former use (14.7), and current use (76.1%). The margin of error on opinion estimates based on the sample of 1,500 is ± 2.53% in a 95% confidence interval.

### Awareness of Precision Medicine

Participants were asked, “Have you ever heard of precision medicine?” Of the respondents, 11.5% answered, “I have heard of it and I know what it is”; 58.2% answered, “I have heard of it, but I do not know what it is”; and 30.3% answered, “I have never heard of it.” Among the 1,046 respondents who have heard of “precision medicine,” respondents learned about it through media such as TV and radio (58.9%), the internet (49.8%), magazines and newspapers (21.5%), and hospitals (13.6%).

After reading the definition and a brief example of precision medicine, 96.1% responded that precision medicine is important for prevention and treatment of disease. The higher the education (less than middle school [89.1%], high school [95.9%], and college and more [96.4%]) or the higher the household income (<\2,000,000 [94.8%], \2,000,000 – \3,990,000 [95%], \4,000,000 – \5,990,000 [96.1%], and \6,000,000 ≤ [97.9%]) of the respondent, the higher the rating of the importance of precision medicine. Of the 69.9% of respondents who said they did not know what precision medicine was before the survey, 91.5% agreed on the importance of precision medicine.

### Attitude Toward the Need to Implement the RCP-PMR

**FIGURE 1:**
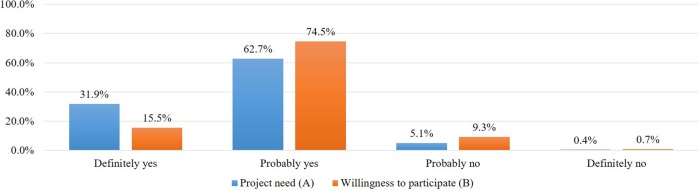
Participants’ attitude toward the need to implement the RCP-PMR **(A)** and their willingness to participate in the study **(B)**.

After introducing the concept of precision medicine, the plan of the project, and data sharing policies, we asked about the need for the RCP-PMR (Figure 1A). Most respondents (94.5%) agreed on the need to implement the study and, among variables, men (95.0%), older adults (older than 50, 96.1%), those with a higher education (high school [95.6%], and college and more [94.6%]), and current SNS users (95.3%) highly supported implementing the study (Table 2).

Adjusting for the other factors in Tables 1, 2 showed no significant differences between genders, region groups, and household income groups by a multiple logistic regression treating the need of the RCP-PMR as a binary independent variable. Younger age (20–29 [*p* = 0.015]) and lower education level (less than middle school [*p* = 0.021]) were independently associated with lower levels of support for the study. The SNS non-user group was significantly associated with higher levels of support for the study (*p* = 0.019).

### Willingness to Participate in the Study

When asked about their intention to participate in the project, 83.5% of respondents said they would participate and 16.5% said they would not (Figure 1B).

Adjusting for the other factors in Table 1, age 20–49 (20–29: *p* = 0.001; 30–39: *p* = 0.002; 40–49: *p* = 0.047) and lower household income (< \2,000,000: *p* < 0.0001; \2,000,000 – \3,990,000: *p* < 0.0001) were independently associated with lower levels of willingness to participate in the study (Table 2). As a group, those who did not have experience with SNS were significantly more likely to say they would participate in the study if asked (*p* = 0.039).

### Concerns About Participating in the Study

Among 247 respondents who said they would not participate in the study, we asked about the reasons why they have no intention to participate in the study. Of the respondents, 94 (38.1%) expressed concerns about it being time consuming or a hassle. The leakage of personal information was a concern for 83 respondents (33.6%), and 62 respondents (25.1%) were concerned about the lack of returning benefits of participation.

### Willingness to Provide Personal Information and Samples for the Study

**FIGURE 2:**
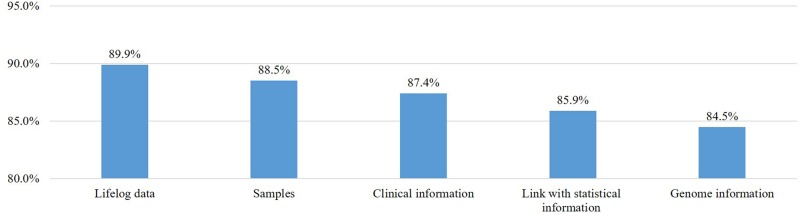
Willingness to provide personal information and samples if asked.

We asked all respondents, including those who said they would not participate, about their willingness to provide various types of samples and data to this project (Figure 2). Most respondents replied that they would provide clinical information (*n* = 1,311, 87.4%), samples (*n* = 1,328, 88.5%), genetic information (*n* = 1,268, 84.5%), and data on lifestyle (*n* = 1,349, 89.9%) and would link their data with existing national statistics from the Meteorological Administration and the Ministry of Environment (85.9%). Many of the respondents who would not provide specimens or personal health information were concerned about personal information leakage and privacy violations. Most respondents had a positive attitude toward providing specimens and information, and 84.5% to 89.9% of participants said they would provide certain types of samples and information.

### Appropriate Research Institute to Undertake the Study

We asked all respondents about their opinion on what type of research institute would be suitable in initiating the RCP-PMR. The majority of respondents (66.9%) said that it should be carried out by government research institutes. Less than 20% of respondents agreed that the study should be undertaken by government-funded research institutes (19.7%), other non-profit institutes (8.8%), and industry and private research institutes (4.5%).

### Using the Collected Samples and Personal Information

**FIGURE 3:**
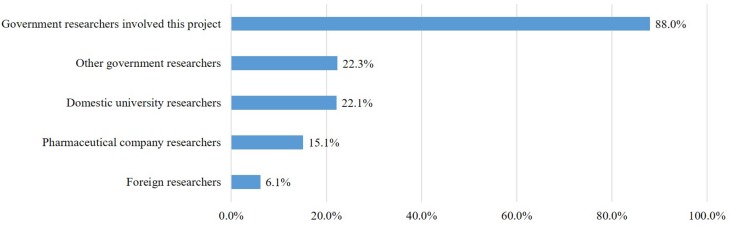
Willingness to share personal information and samples with researchers.

The RCP-PMR plans to authorize qualified researchers to use data and specimens collected from cohort participants to perform various research activities. In the questionnaire, we asked about their willingness regarding the range of researchers allowed to use the personal information and samples provided by participants. Most respondents responded negatively to their specimens and information being used by researchers who are not directly involved in the RCP-PMR. The approval rate for their own data being used by the government researchers running this project was quite high (88%), but the approval rates for its use by other government researchers (22.3%), domestic university researchers (22.1%), pharmaceutical researchers (15.1%), and foreign researchers (6.1%) were all low (Figure 3).

### Participation Benefits

**FIGURE 4:**
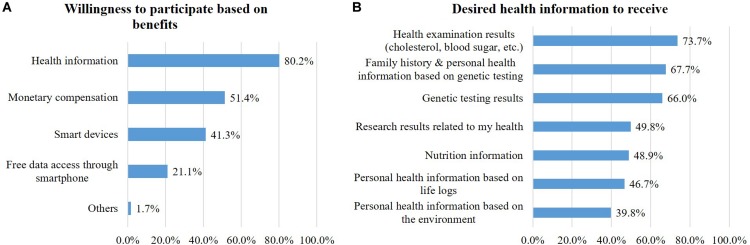
Participation benefits: Willingness to participate based on benefits **(A)** and desired health information to receive **(B)**.

Respondents were asked about the importance of incentives behind their decision of whether to participate. Respondents said incentives are very important (31.4%) or rather important (61.0%). The incentives for participation were receiving health information (80.2%), monetary compensation less than 50,000 KRW (about 42 USD) per year (51.4%), and smart devices (41.3%) (Figure 4A). When asked about the information they wanted to receive, laboratory results (cholesterol, blood sugar, etc.) was the highest (73.7%), followed by health information based on family history and genetic testing (67.7%), genetic testing results (66%), health-related research results that use their own information (49.8%), nutrition information (48.9%), health information based on lifelog results (46.7%), and environment-based health information (38.9%) (Figure 4B).

### Participation in Decision-Making

**FIGURE 5:**
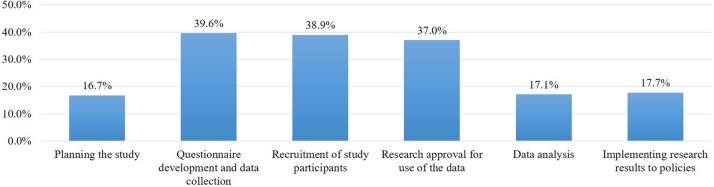
Desired participation in decision making of the study by phase.

In large-scale cohort projects that receive a variety of samples and information, it is important to communicate with participants to identify stakeholder needs. Participants were asked whether the opportunity to comment on the project design or operation was important or not, and most respondents (89.7%) said it was important. When we subsequently asked about the phases of the study in which they wanted to participate in decision-making, the rates of participation in each phase were all under 40%. They mainly wanted to be involved in three phases: the questionnaire development and design of personal data collection (39.6%), study participant recruitment (38.9%), and deciding which research projects will use the collected data (37.0%) (Figure 5).

### Change in Perception Regarding Project Participation

We expected that participants who completed the survey would have a better understanding of the project, and this was assumed to have an impact on their willingness to participate in the project. In order to confirm the change in perception regarding project participation, near the end of the survey, respondents were once again asked, “Would you participate in the project?” Of the respondents, 83.5% said they would participate in the project, and their intention to participate in the project was almost unchanged from the beginning to the end of the survey.

## Discussion

A large-scale research cohort study is important to ensure that the RCP-PMR develops in a manner that respects public values and interests as well as the instrumental goals of recruiting and retaining participants. We conducted a survey of Korean adults who are potential participants to understand their opinions on the RCP-PMR. Five points of discussion regarding the results analysis are presented below.

The first point is regarding the public attitude toward the citizen participation cohort model for precision medicine. Our survey results showed that 94.5% of participants answered that we need this project, which is 15.5% higher than the Kaufman et al. (2016) study in the United States (Kaufman et al., 2016). The intention to participate is very high (83.5%), which is much higher than that of the Kaufman et al. study (54%) (Kaufman et al., 2016). We can extrapolate that this result is because of the sense of the nationalism of and the familiarity with cutting-edge technology among Koreans. Kim (2007) showed that the majority of participants had very positive attitudes toward the life sciences industry as the most important pillar of the South Korean economy (Kim, 2007) and Okita et al. explained that a sense of responsibility to their families and society may have a positive impact on increasing the willingness to participate in genomics research in Japan (Okita et al., 2018). Bak and Kim (2016) also provided evidence that the higher the trust in scientific expertise, the higher the level of public support for social problem-solving research in South Korea (Bak and Kim, 2016). This positive attitude of the general public also corresponds with the Ishiyama et al. (2008) finding that 69.4% of Japanese participants favored the promotion of genomic studies related to medicine (Ishiyama et al., 2008).

More specifically, this result also showed that a lower level of support for the project is associated with lower education levels, consistent with Okita et al. in Japan and a focus group study in China (Chen et al., 2013; Okita et al., 2018). To establish strategies to recruit and retain participants in the research program, it is essential to obtain more evidence on prejudice against or misconceptions of this project results from low levels of genomic literacy and to explore favorable ways of knowledge translation and transfer in order to identify appropriate health and educational interventions (Etchegary et al., 2013; Nakamura et al., 2017). On the other hand, those who do not have experience with SNS were significantly more likely to agree on the need to implement the study and answered that they would participate in the study if asked. While SNS was known as a potential source for participant recruitment and research data for those who are supportive toward this kind of large-scale precision medicine project (Reaves and Bianchi, 2013), this survey result requires more elaborative strategy to encourage them to use SNS and to improve their knowledge about SNS. The evidence can also support the development of educational and awareness programs to familiarize people with genomics and health beyond the scope of the RCP-PMR (Reaves and Bianchi, 2013).

Second, 24.7% of the respondents who were not willing to participate said they would get involved if they had better protection from privacy leakage and if personalized health services were provided. Gaskell et al. in Europe provided a possible reason for participants’ privacy concern that people explicitly expressed that privacy violations are an issue not only in biobanks but also in wider society (Dorey et al., 2018). A recent South Korean governmental report also supports the finding of Gaskell et al. – 4.6% of 4,000 internet users experienced information security incidents, and 97.3% were aware of the importance of personal information security in 2018 (Gaskell et al., 2013). Health information security, such as leakage of personal information in the National Health Insurance Corporation database in the ROK, remains a serious issue (Korean Internet & Security Agency, 2018). Although it is crucial to utilize the RCP-PMR database for data sharing in research and commercial sectors, this emerging public view of privacy protection suggests that a well-established model for privacy protection and communication with the public are needed.

Third, regarding benefits of participation, 80.2% of respondents wanted health information including health examination and genetic testing results as an appropriate compensation for participating in the project. People in South Korea already receive health results from regular medical checkups such as general health exams and cancer screenings, which are covered by the national health insurance program, as they are in Japan, Taiwan, and Singapore. However, the possibility of receiving genetic testing results in particular could be the reason participants preferred a return of health information to other forms of compensation. This public interest in return of results should be specifically implemented in the RCP-PMR.

Next, in terms of public willingness to allow different types of researchers to use their data and samples, a significant gap was found between researchers running the program and researchers outside the program, particularly private companies. This project will be carried out by researchers who are qualified to research various specimens and information provided by the participants. More than 85% of respondents said they would provide a variety of specimens and personal information, and 88.0% of participants agreed that government researchers conducting the project should be able to use their specimens and information. However, they had a negative attitude toward its use by other government researchers, non-government researchers such as private companies, and foreign researchers. The United States survey showed that participants agreed to use various researchers such as researchers at the National Institutes of Health (79%), other government researchers (44%), university researchers in the United States (71%), pharmaceutical or drug company researchers (52%), and university researchers in other countries (39%) (Kaufman et al., 2016). In the ROK, however, only 6.1% – 22.3% of the participants agreed to have their data used by researchers other than the government researchers running the project. This result suggests that sharing the provided samples and information with non-government researchers such as private companies may reduce willingness to participate. For the success of the project, however, it is necessary to communicate with the lay public as well as the RCP-PMR participants to improve their understanding of the purpose of the project and how data sharing can contribute to disease research and prevention.

Finally, in terms of the citizen partnership model in the decision-making process of the RCP-PMR, the findings addressed a gap between the 89.7% of respondents willing to participate generally in the decision-making process of the RCP-PMR and their low response rate of willingness to participate in each individual study phase (16.7–39.6%). We can interpret that researchers in Korea did not consider the engagement of research participants in the decision-making process of biobanking or personalized medicine research, so the lay public were not experienced in how to involve themselves in the decision-making process of this kind of research project. Communication with civil society and patient organizations at various stages of the research would be important in improving understanding and reaching consensus to achieve the goals of precision medicine.

This study also found that around 40% of respondents would want to provide their opinions on questionnaire development and data collection (39.6%), recruitment (38.9%), and approval of research for data use (37.0%). In particular, this finding is consistent with the United States study, which found that the respondents wanted to be involved in helping decide what kinds of research are appropriate (45%) and what to do with the study results (45%) (Kaufman et al., 2016).

## Limitations

The findings of this study have to be seen in light of some important limitations. First, the participants highly supported the RCP-PMR but the results may not reflect actual participation rate of South Korean population. The full response rate from the invitation is 2.9%, so the survey results do not reflect actual willingness to participate. In addition, all invited participants were on panels of a survey firm, so they possibly responded more favorably than the general population.

Second, this survey focused on the social acceptance of the implementation of and participation in the RCP-PMR, meaning that participants’ perceptions of what can be derived from this study were not examined. For instance, ethical concerns over sharing genomic data, including that of the patient’s family members, is an emerging issue in East Asian countries (Johnsson et al., 2010; Yoshizawa et al., 2017). Families have played significant roles in genetic research, and their value is re-illuminated in the era of genomic medicine. It is important to make progress in data sharing while simultaneously protecting the privacy and interests of patients and families and returning its benefits to them (Yoshizawa et al., 2017). More empirical evidence to identify interrelated and cross-cultural factors of the social acceptance of government-led biomedical research is also required.

In addition, public awareness of the risks of health-related data sharing has not been fully investigated in this research. The perceptions of potential risks of data sharing are influenced by attitudes to genomic data sharing (Takashima et al., 2018). The applications of health-related data sharing on the grounds of research and public interest, without due regard for the perspective of patients and the lay public, could run the risk of fostering distrust toward healthcare data collection (Chen et al., 2013). Further studies to investigate the issues, such as discrimination, are essential to promote voluntary participation by the public.

Finally, our finding that lower levels of education population correspond to lower levels of support for the project is possibly associated with genomics-related literacy. However, this study is limited by the fact that the survey participants do not know how much they understand about the project and genomics and responded to the survey based on the guidance given. Thus, as discussed above, we suggest further empirical studies to evaluate genomics-related literacy as a basis of establishing health and educational strategies.

## Conclusion

These survey results will influence the policy development of the precision medicine national cohort program in South Korea. We found that the need for the project, the willingness to participate in the project, and the willingness to provide specimens and information to the project are higher than the results of the United States and Japan. This survey also found a strong willingness of the public to participate in the decision-making process of the RCP-PMR. However, more importantly, this survey also revealed that participants who have negative attitudes toward the RCP-PMR are concerned about privacy violations and the majority of participants disagreed with specimen and data sharing with researchers other than the government researchers who run the project.

For the success of this national project, such findings will determine the public engagement policy for precision medicine in South Korea. As a crucial point, the policy will focus on communicating with the general public and patients how the project and sharing of data with other researchers can help healthcare. This project will also establish a system of the governance that respects the opinions of various stakeholders, including civil society organizations, patient groups, and researchers in the project planning and execution.

## Data Availability Statement

The datasets generated in this study can be accessed from the corresponding author upon reasonable request.

## Ethics Statement

The studies involving human participants were reviewed and approved by Institutional Review Board (IRB) of the Yonsei University. The ethics committee waived the requirement of written informed consent for participation.

## Author Contributions

HK and HRK equally contributed to framing the research design, analyzing the data, and drafting and revising the manuscript. SK contributed to conducting the survey and drafting the manuscript. EK contributed to the interpretation of the findings. SYK participated in the framing of the initial research design and interpretation of the findings. H-YP continuously provided feedback on conducting the survey and revising the manuscript. All the authors read and approved the final manuscript.

## Conflict of Interest

The authors declare that the research was conducted in the absence of any commercial or financial relationships that could be construed as a potential conflict of interest.

## Abbreviations

ELSI, Ethical: Legal and Social Implications
IRB: Institutional Review Board
KCDC: Korea Centers for Disease Control & Prevention
PMI-AURP: Precision Medicine Initiative All of Us Research Program
RCP-PMR: Resource Collection Project for Precision Medicine Research
ROK: Republic of Korea
SNS: Social Networking Sites.

## References

[B1] Alzu’bi A. A. Zhou L. Watzlaf V. J. M. (2019). Genetic variations and precision medicine. *Perspect. Health Inf. Manag.* 16:1a. PMC6462879 31019429

[B2] Bak H. J. Kim M. (2016). The relationship between public support for scientific research and political orientations: the case of research for social problem-solving. (in Korean). *J. Technol. Innov.* 24 107–137. 10.14383/SIME.2016.24.3.107

[B3] Chen H. Gottweis H. Starkbaum J. (2013). Public perceptions of biobanks in China: a focus group study. *Biopreserv. Biobank.* 11 267–271. 10.1089/bio.2013.0016 24835257

[B4] Collins F. S. Varmus H. (2015). A new initiative on precision medicine. *N. Engl. J. Med.* 372 793–795. 10.1056/NEJMp1500523 25635347 PMC5101938

[B5] Dorey C. M. Baumann H. Biller-Andorno N. (2018). Patients data and patient rights: swiss healthcare stakeholders’ ethical awareness regarding large patient data sets - a qualitative study. *BMC Med. Ethics* 19:20. 10.1186/s12910-018-0261-x 29514635 PMC5842517

[B6] Etchegary H. Green J. Dicks E. Pullman D. Street C. Parfrey P. (2013). Consulting the community: public expectations and attitudes about genetics research. *Eur. J. Hum. Genet.* 21 1338–1343. 10.1038/ejhg.2013.64 23591403 PMC3831079

[B7] Gaskell G. Gottweis H. Starkbaum J. Gerber M. M. Broerse J. Gottweis U. (2013). Publics and biobanks: pan-European diversity and the challenge of responsible innovation. *Eur. J. Hum. Genet.* 21 14–20. 10.1038/ejhg.2012.104 22669414 PMC3522201

[B8] Genetics Home Reference (2019). *What is Precision Medicine?* Available online at: https://ghr.nlm.nih.gov/primer/precisionmedicine/definition (accessed June 1, 2019).

[B9] Genomics England (2019). *The 100,000 Genome Project.* Available online at: http://www.genomicsengland.co.uk/ (accessed June 1, 2019).

[B10] Haga S. B. (2017). Update: looking beyond the 100,000 Genome Project. *Per. Med.* 14 85–87. 10.2217/pme-2016-0101 29754551

[B11] Ishiyama I. Nagai A. Muto K. Tamakoshi A. Kokado M. Mimura K. (2008). Relationship between public attitudes toward genomic studies related to medicine and their level of genomic literacy in Japan. *Am. J. Med. Genet.* 146 1696–1706. 10.1002/ajmg.a.32322 18546279

[B12] Johnsson L. Helgesson G. Rafnar T. Halldorsdottir I. Chia K. S. Eriksson S. (2010). Hypothetical and factual willingness to participate in biobank research. *Eur. J. Hum. Genet.* 18 1261–1264. 10.1038/ejhg.2010.106 20648060 PMC2987483

[B13] Kaufman D. J. Baker R. Milner L. C. Devaney S. Hudson K. L. A. (2016). Survey of U.S adults’ opinions about conduct of a nationwide precision medicine initiative^®^ cohort study of genes and environment. *PLoS One* 11:e0160461. 10.1371/journal.pone.0160461 27532667 PMC4988644

[B14] Kim H. Kim S. Y. Joly Y. (2018). South Korea: in the midst of a privacy reform centered on data sharing. *Hum. Genet.* 136 627–635. 10.1007/s00439-018-1920-1 30121900 PMC6132641

[B15] Kim J. S. (2007). A survey research on the social and ethical implication of life science and regenerative medicine. (in Korean). *J. Health Med. Sociol.* 21 157–196.

[B16] Korean Internet & Security Agency (2018). *2017 Survey on Information Security - Summary Report. [Internet]. Ministry of Science and ICT.* Available online at: https://www.kisa.or.kr/eng/usefulreport/surveyReport_List.jsp (accessed August 20, 2019).

[B17] Nakamura S. Narimatsu H. Katayama K. Sho R. Yoshioka T. Fukao A. (2017). Effect of genomics-related literacy on non-communicable diseases. *J. Hum. Genet.* 62 839–846. 10.1038/jhg.2017.50 28490765

[B18] National Institutes of Health (2019). *All of US Research Program.* Available online at: http://www.allofus.nih.gov (accessed June 2, 2019).

[B19] Okita T. Ohashi N. Kabata D. Shintani A. Kato K. (2018). Public attitudes in Japan toward participation in whole genome sequencing studies. *Hum. Genomics* 12:21. 10.1186/s40246-018-0153-7 29653595 PMC5899336

[B20] Peplow M. (2016). The 100,000 genomes project. *BMJ* 353:i1757. 10.1136/bmj.i1757 27075170

[B21] Reaves A. C. Bianchi D. W. (2013). The role of social networking sites in medical genetics research. *AJMG* 161 951–957. 10.1002/ajmg.a.35903 23554131

[B22] Takashima K. Maru Y. Mori S. Mano H. Noda T. Muto K. (2018). Ethical concerns on sharing genomic data including patients’ family members. *BMC Med. Ethics* 19:61. 10.1186/s12910-018-0310-5 29914459 PMC6006763

[B23] Yoshizawa G. Sasongko T. H. Ho C. H. Kato K. (2017). Social and communicative functions of informed consent forms in East Asia and beyond. *Front. Genet.* 8:99. 10.3389/fgene.2017.00099 28775738 PMC5517404

