# Supplementary material for: Public Attitudes Toward Precision Medicine: A Nationwide Survey on Developing a National Cohort Program for Citizen Participation in the Republic of Korea
Source: Front Genet. 2020 May 12;11:283. doi: 10.3389/fgene.2020.00283 (PMC7235362; doi:10.3389/fgene.2020.00283)
Supplement: Supplementary file 1 [file Data_Sheet_1.pdf]

Supplementary Material

Questionnaire

Title: National Survey on the Resource Collection Project for Precision Medicine Research[1]

1 Eligibility of participants

SQ1. How old are you?

① 20-29
② 30-39
③ 40-49
④ 50-59
⑤ 60

SQ2. What is your gender?

① Men
② Women

SQ3. What do you live now?[2]

① Seoul
② Gyeonggi/Incheon/Gangwon
③ Daejeon/Chungcheong/Sejong
④ Daegu/Gyeongbuk
⑤ Busan/Kyongnam/Ulsan
⑥ Kwangju/Jeolla/Jeju

---

[1] Translation from the original text in Korean. The original title in Korean reads 『'정밀의료 연구자원 확보 사업'에 대한 국민 설문조사』.

[2] We asked 16 provisions applying the regional quarters and integrated the choices into 6 regions as shown.

2 Perception of the "Resource Collection Project for Precision Medicine Research (RCP-PMR)"

Q1. Have you ever heard of "precision medicine"?

⑦ I have heard of it and I know what it is. (☞Move to Q1.1)

⑧ I have heard of it, but I don't know what it is. (☞Move to Q1.1)

⑨ I have never heard of it. (☞Move to Q2)

Q1.1. If you have heard of "precision medicine," how did you hear about it? (please select all that apply)

① Through media (TV, radio, etc.)
② Through the internet
③ Through magazines or newspapers
④ At a hospital
⑤ Other (please specify)

※ The box below provides an introduction to "precision medicine." Please read the sentences in the box carefully and answer the following questions.

○ "Precision medicine" refers **<u>to providing personalized healthcare service, such as personally optimized disease prevention, drug treatments, or interventions</u>**, considering the diverse genetic/environmental factors, disease history, lifestyle, etc. of each person.

- For example, if a patient has the gene for an adverse effect (e.g. drug resistance) to a specific drug, a prescription for another drug for the same disease can prevent the adverse effect and increase treatment efficacy.

- Effects of the same cancer drug treatment vary from person to person, such as no treatment effect or severe adverse effect. However, if precision medicine is applied, the patient's clinical data and genome data are analyzed to prescribe the proper drug, increasing the treatment's effect.

-Precision medicine is also used for disease prediction. For example, American actor Angelina Jolie found out she was at high risk for breast cancer due to family history and the breast cancer gene. She had preventive surgery to avoid breast cancer. Furthermore, precision medicine can predict chronic diseases such as cerebral infarction and diabetes based on genome and lifestyle

information, as well as prevent and manage diseases through proper adjustments in lifestyle and diet.

Q2. How important do you think “precision medicine” is for preventing and treating diseases?

① Very important
② Important
③ Unimportant
④ Very unimportant

Q3. If precision medicine becomes widely used, in which area do you think it will be the most helpful?

① Disease prevention service (e.g. prevention of chronic diseases)
② Personalized clinical service (e.g. early diagnosis of genetic disease and drug selection)
③ Expansion of healthy life time
④ Decreasing medical expenses
⑤ Don’t know
⑥ Other (please specify)

※ The box below provides an introduction to the RCP-PMR. Please read the sentences in the box carefully and answer the following questions.

○ The RCP-PMR is a project to collect specimens (e.g. blood), clinical data, lifestyle/environmental information, and genome information that are necessary for research and technological development from persons who agree to participate in the project.

Developed regions such as the United States, Europe, and Japan actively carry out projects to collect research resources to make precision medicine possible and improve regulations and policies related to precision medicine.

○ Personal information that is collected from this project will be stored and managed in anonymized forms (deleting names and personally identifiable information and substituting it with a combination of numbers and letters) to prevent the tracking or re-identification of participants under the *Personal Information Protection Act*.

◯ Only qualified researchers can use the anonymized data for researching the interrelations between genetics, the environment, and lifestyle with health and disease. Researchers will study diverse diseases using the specimens and datasets.

Q4. Do you think the RCP-PMR should be done?

① Definitely yes
② Probably yes
③ Probably no
④ Definitely no

Q5. If the RCP-PMR proceeds, which institution do you think is appropriate for conducting the project?

① Government or national/public research institutions
② Government-funded research institutions
③ Other non-profit institutions (foundations, academic associations, etc.)
④ Industry/private research institutions
⑤ Others (please specify)

Q6. Would you be willing to participate in the RCP-PMR?

① I would definitely participate in it.
② I would probably participate in it.
③ I would probably not participate in it.
④ I would definitely not participate in it.

Q6.1. (only Q6 ③,④ responders) What is the main reason why you are not willing to participate in the project?

① Because I have concerns about the protection of personal information (☞Move to Q6.1.1)
② Because I would not receive benefits or incentives (☞Move to Q6.1.2)
③ Because I have no time and the participation seems to be inconvenient (☞Move to Q6.1.3)

④ Other (please specify) (☞Move to Q7)

Q6.1.1. If this country protects your personal information against data leakage, would you participate in this project? (☞Move to Q7)

① Yes, I would.
② No, I would not.

Q6.1.2. If you are provided a health service (e.g. genetic testing results) that is useful for disease diagnosis and personalized treatment, would you participate in this project? (☞Move to Q7)

① Yes, I would.
② No, I would not.

Q6.1.3. How much time do you think is appropriate to spend on the survey and health examination necessary for this project? (☞Move to Q7)

Less than □□□ minutes

Q7. Would you participate in the RCP-PMR if you are provided an additional health examination service when you receive the regular health examination that is covered by the National Health Insurance Corporation?

① I would definitely participate in it.
② I would probably participate in it.
③ I would probably not participate in it.
④ I would definitely not participate in it.

Q7.1. (only Q7 ①,② responders) If you participate in the project, which healthcare service would you want to be provided?

① A precise examination such as a comprehensive cancer screening
② Genetic testing results
③ Health management service helpful for preventing diseases
④ Other (please specify)

Q7.2. (only Q7 ③,④ responders) Please describe the reasons why you are not willing to participate in the project.

( )

※ From now on, please answer the questions with the assumption that you will participate in the RCP-PMR.

Q8. The RCP-PMR requires personal clinical information for research that aims to examine the causes, diagnosis, and prevention of diseases. Will you provide your clinical data?

① Yes, I will provide it. (☞Move to Q8.1)

② No, I will not provide it. (☞Move to Q8.2)

Q8.1. (only Q8 ① responders) Please select all the types of clinical information you are willing to provide to this project.

① Clinical test results

② Electronic clinical records from medical institutions (e.g. hospitals)

③ Health examination results and clinical information in the National Health Insurance Corporation

④ Health claim data from the Health Review & Assessment Service

Q8.2. (only Q8 ② responders) Please describe the reasons why you are not willing to provide your clinical information to the project.

( )

Q9. The RCP-PMR requires personal specimens (e.g. blood, saliva, urine, and nail clippings). Will you provide specimens?

① Yes, I will provide them.

② No, I will not provide them. (☞Move to Q9.1)

Q9.1. (only Q9 ② responders) Please describe the reasons why you are not willing to provide specimens to the project.

( )

Q10. The RCP-PMR requires genes that are related to disease onset, diagnosis, and prevention. This project intends to acquire the person's genome through the analysis of the provided specimen. Will you provide your genome information?

① Yes, I will provide it.
② No, I will not provide it. (☞Move to Q10.1)

Q10.1. (only Q10 ② responders) Please describe the reasons why you are not willing to provide your genome information to the project.

( )

Q11. The RCP-PMR plans to conduct the research using lifelog data such as heart rate, step count, and physical activity level to identify correlations between lifestyle and disease onset and prognosis. Will you provide lifelog data?

① Yes, I will provide it. (☞Move to Q11.1)
② No, I will not provide it. (☞Move to Q11.2)

Q11.1. (only Q11 ① responders) Will you agree to automatically transmit your lifelog data by smartphone or smart watch to this project?

① Yes, will I agree to.
② No, I will not agree to.

Q11.2. (only Q11 ② responders) Please describe the reasons why you are not willing to provide your lifelog data to the project.

( )

Q12. The RCP-PMR plans to link its database with those of the Meteorological Administration (MA) and the Ministry of Environment (MOE) to study the relationship between the environment and the onset or prognosis of diseases, such as the effect of fine dust. Will you allow the researchers to link your data with statistics from the MA and the MOE?

① Yes, I will allow it. (☞Move to Q13)

② No, I will not allow it. (☞Move to Q12.1)

Q12.1. (only Q12 ② responders) Please describe the reasons why you are not willing to allow it.

( )

Q13. If you join the RCP-PMR, what types of the following researchers will you allow to use your data? The government would anonymize the collected data for research use so that your data will not be identifiable. Please select all that apply.

① Government researchers who are conducting the RCP-PMR
② Other government researchers
③ Domestic university researchers
④ Pharmaceutical company researchers
⑤ Researchers in other countries

Q14. When deciding whether or not to participate, how important are benefits and incentives?

① Very important
② Somewhat important
③ Not very important
④ Not important at all

Q14.1. If you participate in this project, what incentives do you think are appropriate to receive? Please select all that apply.

① My health information (e.g. health examination results, genetic testing results, etc.)
② Monetary compensation (less than KRW 50,000) [≒ USD 41]
③ A free smart device (e.g. wearable tracker)
④ A free data plan for smartphones
⑤ Other (please specify)

Q15. If you participate in this project, what types of information would you like to receive? Please select all that apply.

① Health examination results (cholesterol, blood sugar level, etc.)
② Genetic testing results
③ Health information based on family history and genes

④ Nutrition information (body mass index, dietary analysis, etc.)
⑤ Health information based on lifelogs
⑥ Health information based on the environment (fine dust, etc.)
⑦ Research results related to my health

Q16. If you had chances to suggest your opinion, do you think it would be important to be able to give your opinions as the RCP- PMR is planned and conducted?

① Very important
② Somewhat important
③ Not very important
④ Not important at all

Q16.1. If you had chances to suggest your opinion, in which phase would you participate to plan and conduct the RCP-PMR?

① Planning the study
② Deciding on the questionnaire development and data collection
③ Deciding on participants' recruitment
④ Deciding which research will use the data
⑤ Analyzing the collected data
⑥ Implementing the research results to policies

Q17. [As you have had a chance to think about the RCP-PMR] Would you participate in this project?

① Definitely yes
② Probably yes
③ Probably no
④ Definitely no

Q17.1. (only Q17 ③,④ responders) Please describe the reasons why you would not participate in it.

( )
